# Successful fat-only whole breast reconstruction using cultured mature adipocytes and conditioned medium containing MCP-1

**DOI:** 10.1038/s41598-023-45169-1

**Published:** 2023-11-03

**Authors:** Hiroko Yanaga, Mika Koga, Hiromichi Nishina, Yoshio Tanaka, Katsu Yanaga

**Affiliations:** 1 Yanaga Clinic and Tissue Culture Laboratory, 1-2-12 Tenjin, Chuo-ku, Fukuoka, 810-0001 Japan; 2 Department of Plastic and Reconstructive Surgery, Faculty of Medicine/Graduate School of Medicine, Kagawa University, 1750-1 Ikenobe, Miki-cho, Kida-gun, Kagawa, 761-0793 Japan

**Keywords:** Regenerative medicine, Tissue engineering

## Abstract

A mastectomy is a curative treatment for breast cancer. It causes breast and soft tissue deficits, resulting in a chest with poor vascularity. Autologous tissue breast reconstruction is commonly associated with donor site morbidity. Breast implants are another reconstruction alternative, but they are associated with infection, rupture, and the need for replacement. Autologous aspirated fat grafting has appeared as an ideal breast reconstruction method, but low graft viability and high resorption remain as the main shortcomings. We developed a novel method for fat-only grafts using cultured mature adipocytes (CMAs) mixed with their condition medium. Twenty-five mastectomy patients, aged 32–72 years, received a mixed grafting of CMAs, MCP1-containing condition medium, and fat grafts for total breast reconstruction. In follow-up periods of 24–75 months, MRI analysis showed full thickness fat-engraftment. The cell proliferation marker Ki67 was negative in post-transplant biopsy specimens from all patients. Aesthetic full breast morphology was achieved, patient satisfaction was evaluated 1 year and 3–6 years after surgery. All grafts were confirmed safe, demonstrating high reliability and long-term sustainability.

## Introduction

The incidence of breast cancer has been increasing worldwide in recent years^1,2^. A mastectomy is a curative treatment for breast cancer, but can leave patients with pain and abnormal feeling due to contracture and can have a negative impact on the quality of life of patients^1,2^. Although autologous tissue flaps or artificial breast implants are commonly used for breast reconstruction after a mastectomy^3–5^, autologous tissue flaps are surgically invasive, and tissue is widely lost at the donor site^4,5^. As such, reconstruction using breast implants is more popular^5–7^, despite being associated with complications caused by the artificial material (infection, capsule contracture, and rupture) and the need to be replaced after several years^6,7^. Recently, the risk of developing breast implant-associated anaplastic large cell lymphoma (BIA-ALCL)^8,9^ has also been reported. The number of patients trying to avoid the risks associated with breast implants has increased, and new treatments are being explored. Recently, aspirated fat grafts have attracted attention as an ideal breast reconstruction method with minimum invasive surgery and less donor site morbidity^10,11^. However, an optimal method for fat grafts is yet to be established. This is because a mastectomy involves the removal of a large amount of tissue, which in turn requires an equally large amount of fat filling to correct the defect. In addition, poor vascular distribution at the recipient site makes it difficult for the fat graft to survive.

While fat grafting is a very useful tool in the surgeon's options, a major problem is the unpredictable degree of resorption and volume loss, with reported survival rates ranging from 20 to 90%^12–15^. It is also known that after fat grafting, necrotic fat grafts are often recognized as foreign bodies and cause absorption, calcification, lipo-necrotic cysts, and abscesses^16–19^.

Cell-assisted lipo-transfer (CAL), on the other hand, has been used as a method to increase the engraftment rate for transplanting adipose tissue with small vessel fraction (SVF) containing adipose-derived stem cells (ASCs)^20–22^. In systematic reviews of CAL to the breast, graft retention varied in the range of 40–80% within one year, and complications were at a rate of 37%^22–25^.

Although the focus has hitherto been on increasing blood vessels because blood flow is required for fat graft survival, we hypothesized that the role macrophages play in angiogenesis and adipogenesis would be the key factor for the survival of fat grafts. Macrophages are required for angiogenesis and adipogenesis, they are the third most important component of adipose tissue and are deeply involved in the regulation of adipose formation^26–28^. It has been reported that maturation is impaired in the fat of SIRT1-deficient mice. This was thought to be due to impaired angiogenesis as well as fewer intra-adipose macrophages. Intravenous injection of a reagent that inhibits macrophages (clodronate liposomes) in normal mice has been shown to reduce macrophages in adipose tissue and inhibit angioplasty^28^. This indicates that macrophages are essential cells for the complete formation of adipose tissue.

In addition, MCP-1 has been reported to be important for increasing macrophage infiltration into adipose tissue^29–31^. This has been demonstrated by the fact that MCP-1 (CCL2) produced in adipose tissue attracts macrophages to tissues in mice lacking the CCR2 receptor^31^. Thus, we decided to use CMAs, which secrete high concentrations of MCP-1 and were expected to further enhance angiogenic and adipogenic potential.

Since 2010, 67 partial breast reconstructions and breast augmentations have been performed with a mixture of CMAs, MCP1-containing conditioned medium, and fat grafts (hereafter referred as CMAM-FGs). The fat engraftment rate of CMAM-FGs was over 90%, and no adverse events occurred. Therefore, CMAM-FGs were applied to whole breast reconstruction after mastectomy, where the efficacy and safety of the treatment were evaluated.

## Results

### Human mature adipocyte culture

**Figure 1:**
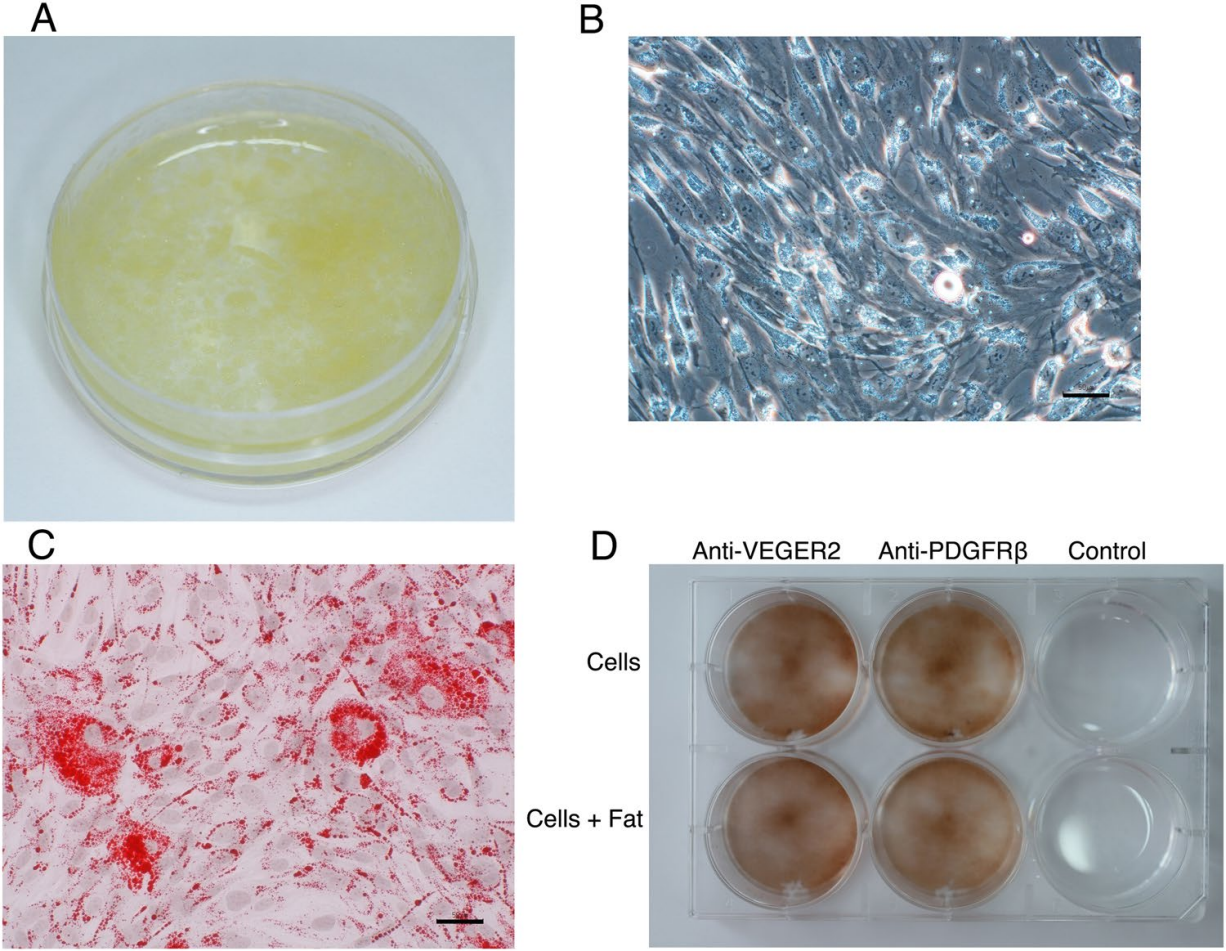
Characterization of cultured mature adipocytes in vitro. (**A**) Images clearly show the state of the microfat. (**B**) Phase-contrast micrographs show the typical elongated adipocytes contained a large amount of fat granules. (**C**) Adipocytes are stained red with Sudan III, indicating that mature adipocytes incorporate fat granules into the cells. (**D**) Expression of VEGFR2 and PDGFRβ in CMAs. CMAs that had been passaged twice were seeded in 1 × 10^4^ cells/6 wells (9.6 cm^2^/well). A CMAs-only group and a CMAs + aspirated fat graft mixed group (1:4) were prepared and cultured for 5 days. VEGFR2 and PDGFRβ immunohistochemical staining was conducted. Both groups strongly expressed VEGFR2 and PDGFRβ. Control: Goat primary antibody (negative).

The subjects of this study were 25 patients who had previously undergone a mastectomy. Approximately 3 cc of microfat was harvested from the patient’s lower abdomen by subcutaneous aspiration (Fig. 1A). Human mature adipocytes (2–3 × 10^6^ cells) were isolated from 2.8 ± 0.9 cc and were cultured at a density of approximately 4 to 5 × 10^4^/ cm^2^. The total number of transplanted cells in the clinical application was 884.20 × 10^7^, with a mean value of 17.00 × 10^7^ ± 3.09 × 10^7^. (The total number of transplanted cells in the first application was 395.02 × 10^7^ with a mean of 15.80 × 10^7^ ± 2.33 × 10^7^, the total number of transplanted cells in the second application was 446.28 × 10^7^ with a mean of 17.85 × 10^7^ ± 3.31 × 10^7^, and the third application was only performed in two patients, bringing the total number of transplanted cells to 42.90 × 10^7^ with a mean of 21.45 × 10^7^ ± 0).

Cultured mature adipocytes retained a large amount of fat granules that appeared light in phase contrast micrographs (Fig. 1B). Adipocytes were specifically stained red with Sudan III, indicating that mature adipocytes incorporated fat granules (Fig. 1C).

### Cultured mature adipocytes (CMAs) expressed VEGFR2 and PDGFRβ in immuno-histological analysis in vitro

Both CMAs alone and CMAs plus aspirated fat graft yielded results showing the presence of cells with strong vascular endothelial growth factor receptor 2 (VEGFR2) and platelet derived growth factor receptor-beta (PDGFRβ) expression (Fig. 1D). VEGFR2 is a protein expressed only in vascular endothelial cells, and its progenitor cells are the primary responders to VEGF signaling, indicating that blood vessels were being formed. Peroxisome proliferator-activated receptor-γ (PPARγ) is mostly expressed in white adipose tissue, where it works as one of the master regulators of adipogenesis^32^. PDGFRβ is expressed when its PPARγ in adipose progenitor cells is stimulated by fatty acids or other substances. Therefore, the presence of cells with strong PDGFRβ expressions showed that they included adipose progenitor cells.

### Cell-surface marker characterization of CMAs and SVF cells

**Figure 2:**
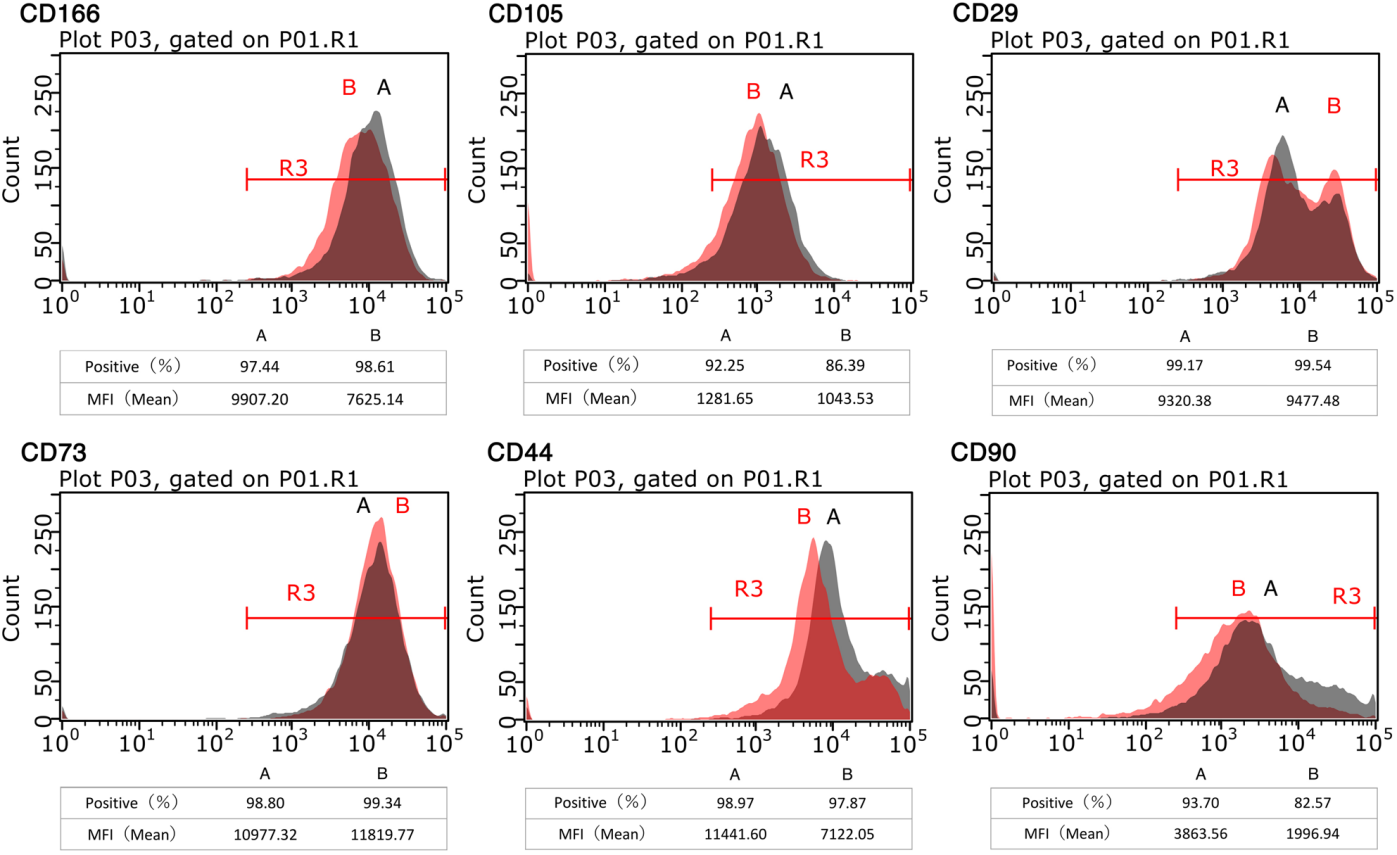
Flow cytometry analysis of the expression of cell surface markers related to various stem cells on SVFs (left) and CMAs (right). IC: Isotype control antibodies were used for control samples. CMAs showed higher expression levels of CD44 and CD90 than observed in SVFs (the figure representing format of Contour plots with outliers is shown in Fig. S2).

We performed flow cytometry to characterize the cell-surface marker profile of CMA mixed populations. The MSC markers CD44, CD73, CD90, CD105, and CD166 were analyzed (Fig. 2). Most marker expressions of CMAs were similar to those of SVF cells. However, CD90 expression showed significant differences. Higher CD90 expression was observed in CMAs (93.7%, MFI 3863.56) compared with SVF cells (82.47%, MFI 1996.94). For CD44, there was not much difference in the positive rate between the two groups, but more cells in CMAs showed higher expression levels.

### Cytokine/chemokine analysis in vitro

**Figure 3:**
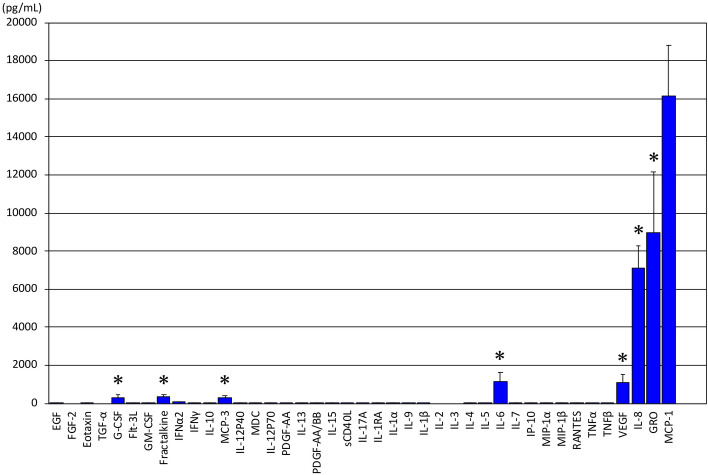
Chemokine/cytokine quantity measurement in condition medium. The concentrations of 41 target proteins in CM(n = 6) were measured using a Milliplex® MAP Cytokine/Chemokine Magnetic Bead Panel kit and the Luminex® system (Merck Millipore). MCP-1 (16.15 ± 2.65 ng/ml) was produced in large quantities, followed by GRO (8.97 ± 3.20 ng/ml), IL-8 (7.09 ± 1.17 ng/ml), and VEGF (1.09 ± 0.43 ng/ml). Production of IL6 (1.17 ± 0.48 ng/ml), MCP-3 (0.29 ± 0.11 ng/ml), fractalkine (0.37 ± 0.07 ng/ml), and G-CSF (0.31 ± 0.13 ng/ml) was also observed. However, only small amounts of other proteins were produced. Indicating values of* p* < 0.01 as compared to the MCP-1.

In CM chemokine/cytokine analysis (n = 6), MCP-1 (16.15 ± 2.65 ng/ml) was produced in the largest amount, followed by GRO (8.97 ± 3.20 ng/ml), IL-8 (7.09 ± 1.17 ng/ml), VEGF (1.09 ± 0.43) ng/ml) and IL6(1.17 ± 0.48 ng/ml) was also produced. Conversely, hardly any TNF-α, IL-1-β, or INF-γ, which cause inflammation, was produced in the CMAs (Fig. 3).

### Cultured mature adipocyte aging examination in vitro

**Figure 4:**
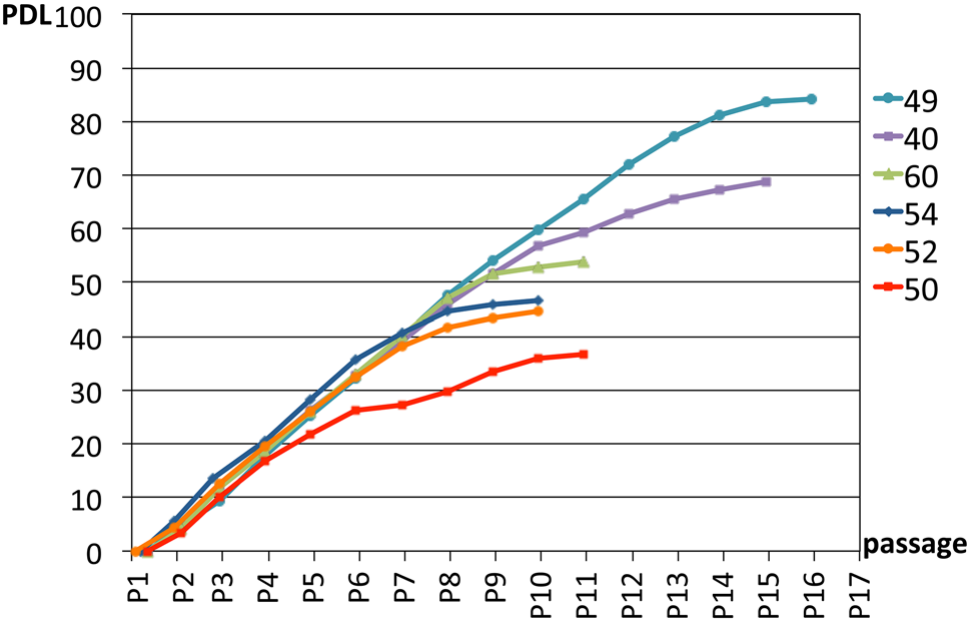
Cultured mature adipocyte aging examination in vitro. This graph shows the number of passages of the adipocytes and the PDLs. Proliferation was confirmed to have stopped due to cell aging. P14 = total 102-day culture: Vertical axis CDL (= PDL). 49 years old (PDL: 84.2, stopped at 102 days), 40 years old (PDL: 68.9, stopped at 95 days), 60 years old (PDL: 54.0, stopped at 67 days), 54 years old (PDL: 46.5, stopped at 61 days) 52 years old (PDL: 44.6, stopped at 62 days), 50 years old (PDL: 36.8, stopped at 67 days).

A population doubling level (PDL) cell aging test was conducted to confirm the safety of the CMAs from six randomly chosen patients. It was confirmed that CMAs were cultured until they stopped growing (PDL: 55.84 ± 17.63) due to cell aging (61–102 days; 75.67 ± 18.00) (Fig. 4). The results showed that CMAs proceeded to terminal differentiation and stopped proliferating, so the possibility of malignant change (tumorigenesis) was not observed.

### Immunofluorescence and immunohistochemical analysis of regenerated fat after the CMAM-FGs in-vivo

**Figure 5:**
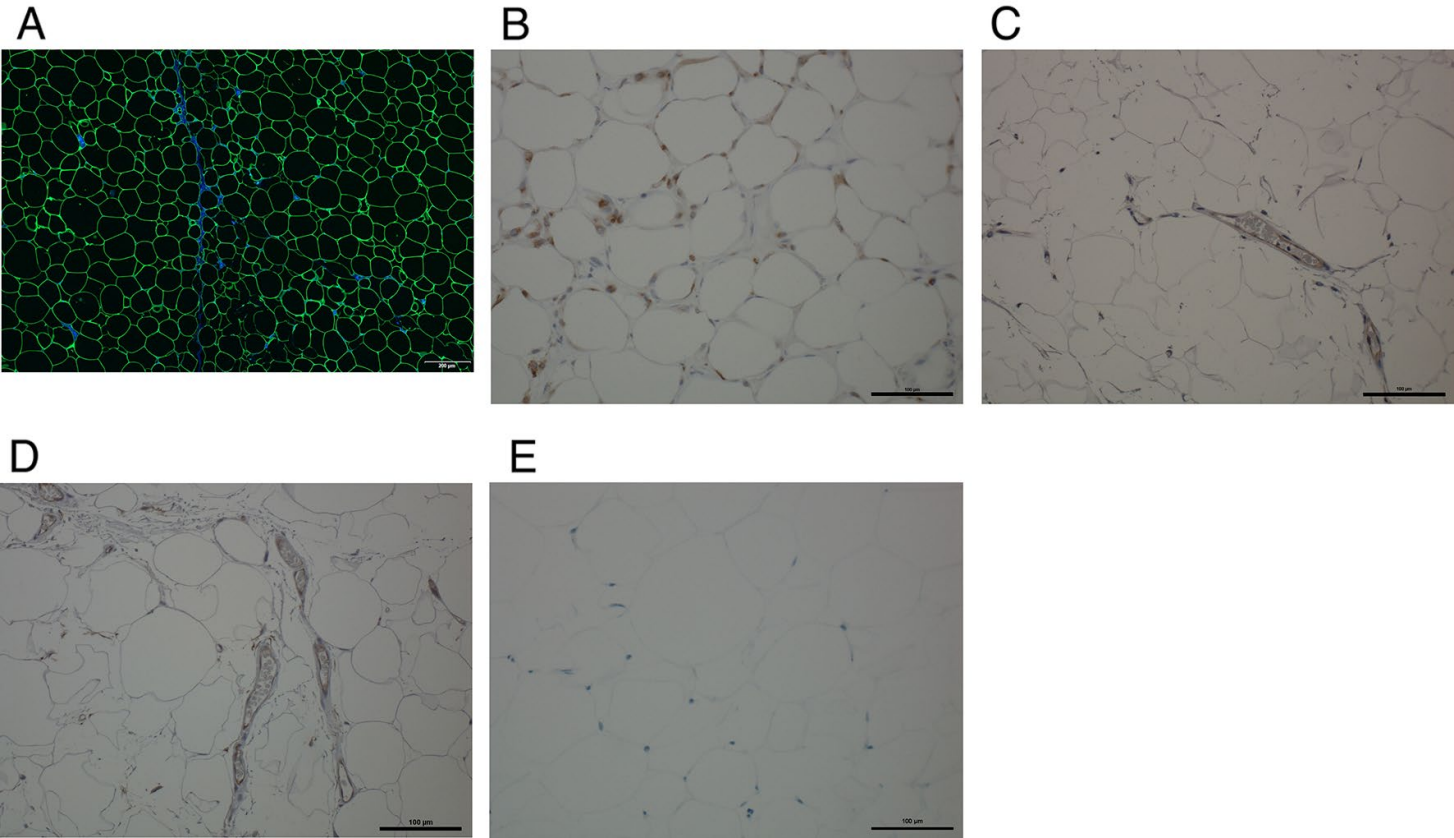
Immunofluorescence and immunohistochemical characterization of regenerated fat 6 months to 2 years after CAM-FG in vivo. (**A**) Anti-perilipin staining shows the adipocyte membrane (green) with DAPI-stained blood vessels and cell nuclei (blue). (**B**) Immunohistochemical detection for macrophage marker CD68 is shown in regenerated adipose tissue. Immunohistochemical detection of endothelial cell markers CD31(**C**) and CD34 (**D**) is shown in vascular remodeling of regenerated adipose tissue. (**E**) Ki67 is not expressed in regenerated adipose tissue. Representative images from n = 6 replicates.

Six months to 2 years after CMAM-FGs were transplanted into the chest by injection, biopsy specimens were stained using anti-perilipin fluorescent immunostaining, which is expressed on the cell membrane of adipocytes (Fig. 5A). The results showed that adipose tissue was formed throughout the reconstructed area. After transplantation, the reconstructed fat was rich in macrophages, which were positive in anti-CD68 staining (Fig. 5B). Anti-CD31 and CD34 immunostaining showed many blood vessels in the regenerated tissue (Fig. 5C,D). Cell proliferation marker Ki67 (Fig. 5E), an important molecular target in the diagnosis of cancer^33^, was negative in the biopsy specimens of all patients.

### Clinical outcomes

The patient summary and clinical results are shown in Table 1. The average engraftment rate of the transplanted CMAM-FGs for the 25 patients was 99.16 ± 4.00%, no postoperative complications were observed in any of the 25 patients. Patients were followed for 2–6 years, and no complications, adverse events or breast cancer recurrences were noted. The graft retention was stable for all patients, and no fat absorption was observed in 24 cases. One patient, who did an intensive exercise program three months after the transplantation, developed a hematoma in the reconstructed breast and absorbed 20% fat. Another patient had a small lump palpated in her reconstructed breast, which was removed and a small cyst found with no evidence of malignancy.

**Table 1:** Total mastectomy patient summary.

Case	Age	Breast cancer stage No. of transplants, volume, cell number	State (months)	MRI full-layer fat formation (horizontal and sagittal sections)	Complications calcification, cysts, fat necrosis	Degree of patient satisfaction* (visual analog scale) One year after surgery
1	51	Left breast, stage I 1st (262 cc)13.36 × 10^7^ cells 2nd (328 cc)17.16 × 10^7^	75	+	None	10
2	48	Left breast, Stage I 1st (147 cc)17.16 × 10^7^ 2nd (360 cc)17.16 × 10^7^	64	+	None	10
3	46	Left breast, stage IIa 1st (273 cc) 17.16 × 10^7^ 2nd (258 cc) 17.16 × 10^7^	57	+	None	10
4	58	Right breast, stage 0 (DCIS) 1st (160 cc) 14.3 × 10^7^ 2nd (293 cc)17.16 × 10^7^	51	+	None	8
5	32	Left breast, stage IIb 1st (196 cc) 11.44 × 10^7^ 2nd (370 cc)14.3 × 10^7^	37	+	Hematoma	8
6	59	Left breast, stage 0(DCIS) 1st (194 cc) 14.3 × 10^7^ 2nd (260 cc) 14.3 × 10^7^	42	+	None	8
7	61	Right breast, stage I 1st (200 cc) 14.3 × 10^7^ 2nd (155 cc) 17.16 × 10^7^	40	+	None	10
8	43	Left breast, stage I 1st (200 cc) 11.87 × 10^7^ 2nd (250 cc) 17.88.45 × 10^7^	39	+	None	8
9	55	Left breast, Stage I 1st (260 cc) 17.16 × 10^7^ 2nd (220 cc) 17.16 × 10^7^	39	+	None	7.6
10	54	Left breast, stage I 1st (340 cc) 14.3 × 10^7^ 2nd (280 cc) 17.16 × 10^7^	38	+	None	10
11	60	Left breast, stage 0 (DCIS) 1st (330 cc) 14.3 × 10^7^ 2nd (277 cc) 17.16 × 10^7^	38	+	Small cyst	8
12	70	Right breast, stage I 1st (248 cc) 12.58 × 10^7^ 2nd (270 cc)17.16 × 10^7^	31	+	None	10
13	68	Left breast, stage IIb 1st (436 cc) 17.16 × 10^7^ 2nd (434 cc) 21.45 × 10^7^	30	+	None	10
14	49	Left breast, stage I 1st (303 cc) 17.16 × 10^7^ 2nd (328 cc) 21.45 × 10^7^	30	+	None	10
15	42	Right breast, stage I 1st (284 cc) 17.16 × 10^7^ 2nd (299 cc) 21.45 × 10^7^	30	+	None	8
16	46	Bilateral breasts, stage I 1st (444 cc) 17.16 × 10^7^ 2nd (367 cc) 21.45 × 10^7^ 3rd (525 cc) 21.45 × 10^7^	30	+	None	10
17	72	Right breast, stage 0 (EAB) 1st (350 cc) 17.16 × 10^7^ 2nd (344 cc) 21.45 × 10^7^	30	+	None	7.5
18	57	Left breast, stage 0 (DCIS) 1st (320 cc) 12.58 × 10^7^ 2nd (240 cc)16.7 × 10^7^	31	+	None	10
19	63	Bilateral breast, Rt stage II Lt Stage 0 (DCIS) 1st (394 cc) 21.45 × 10^7^ 2nd (188 cc) 21.45 × 10^7^ 3rd (288 cc) 21.45 × 10^7^	30	+	None	10
20	32	Left breast, stage 0 (DCIS) 1st (269 cc) 17.16 × 10^7^ 2nd (484 cc) 21.45 × 10^7^	28	+	None	10
21	46	Right breast, stage 0 (DCIS) 1st (148 cc) 17.16 × 10^7^ 2nd (224 cc) 21.45 × 10^7^	28	+	None	10
22	43	Right breast, stage IIb 1st (364 cc) 17.16 × 10^7^ 2nd (306 cc) 13.59 × 10^7^	27	+	None	10
23	58	Right breast, stage IIb 1st (331 cc) 17.16 × 10^7^ 2nd (270 cc) 12.44 × 10^7^	27	+	None	10
24	44	Right breast, stage I 1st (308 cc) 17.16 × 10^7^ 2nd (180 cc) 9.58 × 10^7^	24	+	None	10
25	49	Left breast, stage I 1st (303 cc) 17.16 × 10^7^ 2nd (328 cc) 21.45 × 10^7^	24	+	None	8

The VAS for satisfaction is a horizontal line 10 cm long. At the beginning and at the end, there are two descriptors representing extremes of satisfaction (i.e., no satisfaction and extreme satisfaction). The patients rated their satisfaction by making a vertical mark on the 10 cm line.

*In N^o^ 5, hematoma was observed due to excessive exercise 3 months after surgery. The hematoma healed following fine needle aspiration, but the volume decreased by 20% thereafter.

### Results of MRI image evaluation and patient assessment

**Figure 6:**
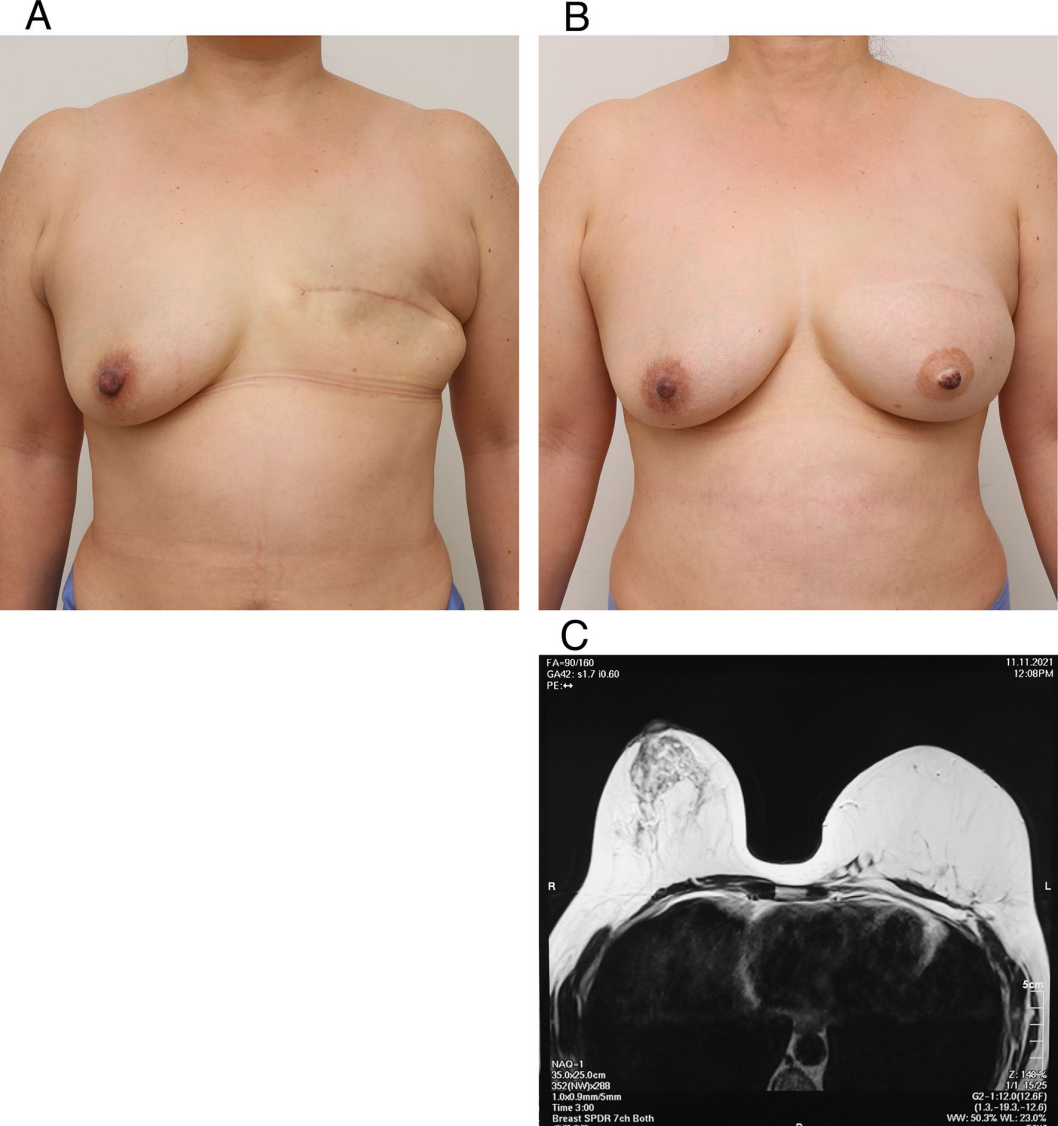
Clinical application of CAM-FG and MRI imaging after transplantation in unilateral case. (**A**) Pre-reconstruction view (after mastectomy) (**B**) Appearance of the breast at 4 years after two CAM-FG applications, 2 years after nipple and areola reconstruction. The nipple and areola were reconstructed using the Yanaga method^49,50^. (**C**) Transverse MRI image 3 years after transplantation. Parenchyma can be seen on the right side, but the left reconstructed side shows only adipose tissue. The adipose tissue shows up as white, and adipose tissue can be confirmed to have formed in all layers. In addition, blood vessels can be seen in the tissue. No fat necrosis, cysts, or calcifications were observed. Stable adipose tissue formation was observed in this or any of the other cases.

**Figure 7:**
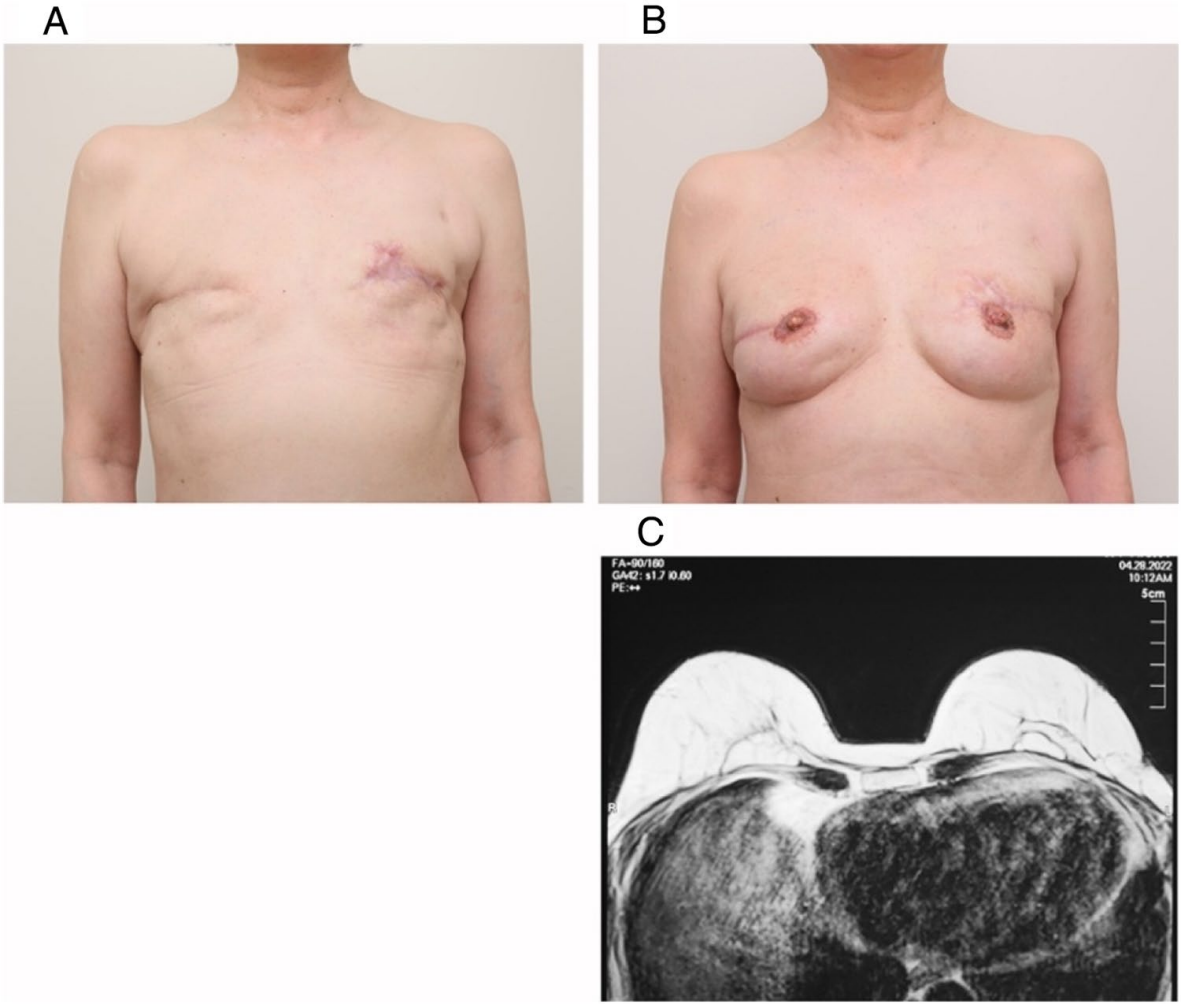
Clinical application of CAM-FG and MRI imaging after transplantation in bilateral mastectomy. (**A**) Pre-reconstruction view (after mastectomy). (**B**) Appearance of the breast at 3 years after 2 applications of CAM-FG. The nipple and areola were reconstructed using the Yanaga method^47,48^. (**C**) Transverse MRI image 2 years after transplantation. No fat necrosis, cysts, or calcifications were observed on either reconstructed side. Stable adipose tissue formation was observed.

The 25 patients treated were examined with MRI imaging annually to evaluate safety. The reconstructed breast after mastectomy showed fat regeneration across the full thickness (Table 1, Figs. 6, 7). The graft retention rate was stable, and no fat necrosis, calcifications, or local cancer recurrence were observed. Naturally shaped reconstructed breasts with soft texture were obtained in all patients. The results of the patient satisfaction survey using the satisfaction visual analog scale (9.24 ± 1.04) one year after surgery are shown in Table 1. In 2023, 3–6 years after surgery, we also evaluated patient satisfaction regarding (1) breast shape (83.3 ± 8.7) (2) breast softness (93.3 ± 6.8) (3) impact on daily activities (88.5 ± 10.5) (4) psychological wellbeing (90.8 ± 10.0) (5) recovery from loss of natural breast (84.2 ± 13.2) (6) general physical wellbeing (85.6 ± 9.4) and (7) surgical results (88.3 ± 11.2).

## Discussion

Successful breast reconstruction using only fat after mastectomy requires a high survival rate and long-term graft retention. Although various advantages of fat grafting have been reported in breast reconstruction, resorption of the fat graft remains an important clinical challenge that needs to be resolved^10–15^. In general, healthy adipose tissue survives because it is supported by a rich vascular network that nourishes it. Naturally, if there are few blood vessels, the adipose tissue will be malnourished and absorbed.

Blood vessels in aspirated fat grafts are crushed by liposuction, so there is no blood flow to the site itself. As a result, the grafts are likely to become necrotic and absorbed after transplantation. Therefore, we thought that the addition of a factor (culture medium) that promotes angiogenesis of fat grafts would improve the fat engraftment rate.

Macrophages are also an important component of adipose tissue and play a key role in angiogenesis and adipogenesis^26–28^. It has also been demonstrated that MCP-1 produced in adipose tissue attracts macrophages to the tissue, and MCP-1 has been reported to be important for increasing macrophage infiltration into adipose tissue^29–31,33–35^. MCP-1 (CCL2), which is produced in adipose tissue, has been demonstrated to attract macrophages to the tissue in mice lacking the CCR2 receptor and by using drugs that inhibit CCR2 signaling^28^. Furthermore, it has been reported that MCP-1 directly binds to human vascular endothelial cells expressing the CCR2 receptor to promote angiogenesis^31^.

We found that the condition medium (CM) secreted by CMAs contained a large amount of MCP-1 (16,000 pg/ml), which attracted and mobilized macrophages in the transplanted fat. This was evidenced by the abundant expression of CD68 in the regenerated fat tissue, demonstrating the presence of macrophages. In addition, since CD31 and CD34 are expressed in the blood vessels in regenerated fat tissue, it can be said that the sequence of fat tissue regeneration, macrophage recruitment, and angiogenesis was induced, which is useful for maintaining the fat formation.

Furthermore, immunostaining of CMAs showed that they contained VEGFR2-positive vascular endothelial progenitor cells and PDGFR-β-positive preadipocytes. PDGFR-β in vascular endothelial cells has been shown to affect angiogenesis^36^. Blocking VEGF signaling with anti-VEGFR2 antibodies prevent adipogenesis, indicating that VEGFR2-positive cells form the final angiogenic adipose^35^ and demonstrating that VEGFR-2-positive cells are required for angiogenesis in transplanted adipose tissue. Because CMAM-FGs contain large amounts of MCP-1 and the vascular progenitor cells mentioned above, we concluded that the vascular network is rapidly restored and macrophages are subsequently mobilized, promoting adipogenesis and resulting in long-term adipose tissue survival.

CMAs secrete the most MCP-1, followed by IL8, IL-6 and VEGF, which are known to be involved in angiogenesis^37–41^. The highest secretion of MCP-1 by our CMAs, which is involved in adipogenesis, may be useful for adipogenesis after the transplantation. CMAs also secrete GRO. GRO is present in normal breast adipose tissue^42^, it is reported to be increased in serum level of obese women and it is associated with adiposity^43^.

In addition, CMAs express very little TNF-α, IL-1β, and INF-γ. Therefore, the mature adipocyte-containing composition obtained by the above method, our CMAM-FGs, have a low potential for causing inflammation and is suitable for adipose tissue maintenance after the transplantation.

Next, we compared ASCs cultured from the SVF fraction without adipocytes with our CMAs, noting that Zwierzina et al. classified SVF cells in human white adipose tissue into three types, all of which also express the mesenchymal stem cell marker CD90 at higher levels as a common feature^44^. Our results show that CMAs cells express approximately tenfold higher levels of CD90 than SVF cells. Although it is possible that SVF could not be completely eliminated from CMAs because they were not enzyme-treated, it is likely that the cells were less damaged; the mixture of adipocytes and SVF may have enhanced their expression compared with SVF-only cells. We also believe that this is because SVF-only eliminates the influence of adipocytes. All of the above indicates that CMA is a distinct cell population from SVF. In addition, CMAs were clearly shown by immunostaining to contain a mixture of both adipose progenitor cells and vascular endothelial progenitor cells, and this mixture may have contributed to the high rate of adipose tissue engraftment and maintenance after transplantation.

It should also be noted that an important factor contributing to the high post-transplantation engraftment rate is the high amount of angiogenic factors secreted by the CMAs, a group of factors that promote adipose tissue retention. Among these, MCP-1 (CCL2), which is released in the largest amount, is essential for complete adipose tissue remodeling because it induces not only angiogenesis but also macrophages necessary for adipose tissue.

We believe that our CMAs cells have achieved long-term survival of transplanted adipose tissue, which is different from conventional adipogenesis, due to the above-mentioned factors.

Cellular senescence studies conducted on six of the 25 patients also showed that the CMAs could be cultured until they stopped growing due to cell aging (PDL: 36.8–84.2, 55.84 ± 17.63, 61–102 days: 75.67 ± 18.00) and had no capacity for sustained cell proliferation or transformation. In addition, the biopsy specimens of all patients after transplantation were negative in testing for Ki67, a cell proliferation marker, suggesting that CMAs is safe.

In a meta-analysis of 21 studies, the estimated number of transplants required to complete whole breast reconstruction was inconsistent, ranging from 2 to 7 sessions^11,15^. In a recent report of 22 cases of total breast reconstruction with fat grafting, 11 cases (50%) abandoned the use of fat only reconstruction and converted to breast implants due to inadequate engraftment^45^. This indicates that engraftment of fat grafts has conventionally been unreliable in whole breast reconstruction until now.

We achieved 100% fat-only whole breast reconstruction with only two transplants for unilateral cases and only three transplants for bilateral cases. This indicates that our grafting method is more stable compared to other fat grafting methods.

Most importantly, the mean engraftment rate was 99.16 ± 4.00%, and graft retention was maintained after 2–6 years, demonstrating greater reliability and sustainability compared to previous fat grafting techniques.

**Figure 8:**
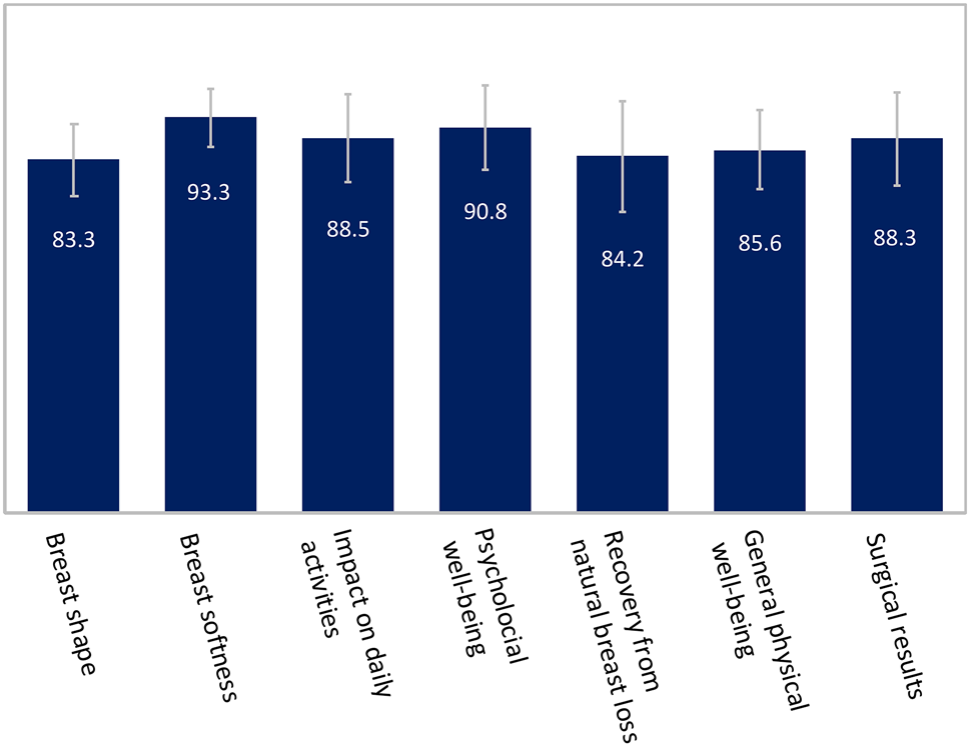
Patient satisfaction 3–6 years after surgery. Satisfaction survey conducted in September 2023 among patients who had undergone surgery 3–6 years earlier. Scale 0–100, 100 expressing the highest level of satisfaction. (1) Breast shape (83.3 ± 8.7) (2) breast softness (93.3 ± 6.8) (3) impact on daily activities (88.5 ± 10.5) (4) psychological wellbeing (90.8 ± 10.0) (5) recovery from natural breast loss (84.2 ± 13.2) (6) general physical wellbeing (85.6 ± 9.4) and (7) surgical results (88.3 ± 11.2).

The reconstructed breast, which is made of fatty tissue as in a healthy breast, has a soft and natural aesthetic appearance, leading to high general patient satisfaction (patient satisfaction VAS: 9.24 ± 1.04) 1 year after surgery. Three to six years after surgery a new patient survey was conducted evaluating satisfaction in terms of breast shape, and softness, patients expressed that breast reconstruction had had a positive impact on their daily activities, as well as psychological and general physical wellbeing. Patients were satisfied with the surgical results and indicated the surgery had helped them recover from the loss of their natural breasts. All seven categories of patient satisfaction stayed above 80% after 3–6 years (Fig. 8). The follow-up periods, to date, are 2–6 years with no complications or recurrence of breast cancer. It is also important in terms of patient safety that the cell proliferation marker Ki67, an important molecular target for cancer diagnosis, was negative in all patient biopsy specimens after transplantation. We plan to continue biopsy testing with patient consent. Further studies with annual MRI examinations are needed to evaluate the long-term clinical efficacy and safety of this procedure.

## Materials and methods

### Patients

This is a retrospective cohort study conducted between December 2015 and December 2022, in which 25 patients with breast cancer who had undergone total mastectomy underwent successful fat-only total breast reconstruction using cultured mature adipocytes and conditioned medium containing MCP-1. This study was approved by the Institutional Review Board of Yanaga Clinic on February, 22, 2015. And then, the study was conducted in accordance with all relevant and applicable governmental and institutional guidelines and regulations, as well as cell therapy guidelines set out by Japanese regenerative medicine law since December, 2015. Yanaga Clinic and Tissue Culture Laboratory, is certified as a Class 2 Regenerative Medicine Cell Processing Facility (registration number: FC7140009; 23 March, 2015) and a Class 2 Regenerative Medicine Provider (registration number for autologous cultured adipocytes transplantation: PB7150005, 11 November, 2015). Clinical outcomes, efficacy, and patient safety were reported as required annually to a government-certified committee on Regenerative Medicine (Gamagori City Hospital Deliberation Committee for Specific Regenerative Medicine as an external committee), the Kyushu office of the Ministry of Health regenerative medicine committee, and finally the Japanese Ministry of Health committee on regenerative medicine. Informed consent was obtained from all participants, and the study protocol conformed to the ethical principles in the Declaration of Helsinki and was approved by the respective institutional review boards.

### Preparation of human mature adipocyte culture and condition medium

Approximately 3 cc of fat was harvested from the patient’s lower abdomen by subcutaneous aspiration, washed, sterilized with antibiotics, digested with collagenase, finely filtered with cell strainer, and centrifuged to collect only the fat component floating in the supernatant. Blood components containing ASCs + SVF precipitated as a pellet in the lower layer were discarded. The microfat particles (Fig. 1A) were cultured in a medium consisting of 10% autologous serum added to α-MEM medium.

From 2.8 ± 0.9 ml of aspirated fat collected, 6.92 × 10^5^ ± 3.25 x 10^5^ adipocytes were isolated. The number of cells obtained at P0 passaging was 10.1 × 10^6^ ± 2.8 × 10^6^, of which 1 × 10^6^ was seeded into one 175 cm^2^ flask and the remaining cells were frozen and stored. The number of cells obtained from P1 passaging was 11.6 × 10^6^ ± 6.2 × 10^6^. Of these, 5 × 10^5^ were seeded per 150 cm^2^ flask. The remaining cells were cryopreserved. The final number of cells seeded in the 150 cm^2^ flasks for transplantation was 4.9 × 10^6^ ± 1.6 × 10^6^. (Fig. 1B).The cell count at final transplantation was 17.00 × 10^7^ ± 3.09 × 10^7^. The process from the cell harvest to transplantation was 122.2 ± 100.18 days. In the final phase, after the cryopreserved cells are thawed, they are cultured and transplanted in a period of 13.3 ± 1.2 days.

The fat droplets of cultured cells were stained with Sudan III (Fig. 1C). All cells collected from patients were labeled and will be stored at the cell processing facility at 1.0 × 10^6^ cells/tube for 10 years.

### Ability of angiogenesis and adipogenesis in CMAs in vitro

One group of CMAs alone and one group of CMAs plus aspirated fat graft were prepared (n = 6) and cultured for five days to determine whether the CMAs contained PDGFRβ (+) preadipocyte cells and VEGFR2 (+) endothelial progenitor cells. Cells in the wells were fixed in 4% paraformaldehyde. The reagents used were anti-VEGFR2 polyclonal antibody (Bioss, 1: 100) as the primary antibody, anti-PDGFRβ polyclonal antibody [Y92]-C-terminal (Abcam, 1:100), goat anti-rabbit IgG H&L (HRP) (Abcam, 1:200) as the secondary antibody, and DAB substrate kit (Funakoshi, SK-4100, [Vector Laboratories, Inc.]) (Fig. 1D).

### Cell surface marker characterization of CMAs

Flow cytometry (Guava easyCyteHT System, easyCyte5HT, S/N: 8470050255, Luminex Corporation) was performed to characterize the cell surface marker profile of human CMA.

Mature adipocytes (n = 6) and SVF cells (n = 6) were isolated and cultured from the same tissue of six individuals. To eliminate individual differences, the mature adipocytes from the six individuals were mixed and cultured in group A and the SVF cells from the same six individuals were cultured in group B. Cells from both groups were detached with trypsin solution and collected. Cells were aliquoted into tubes to make 1 × 10^6^ cells. The cells were immunoassayed with 5 μg antibodies for 30 min at 4 °C listed below, and 10,000 cells were measured in a flow cytometer. Using the measurement results, the expression of MSC markers CD166, CD29, CD105, CD73, CD44, CD90 in both groups were analyzed (Fig. 2).

To calculate CD166, CD29, CD105, CD73, CD44 and CD90 percentage in CMAs and SVFs cells respectively, the analysis was performed as follows (Fig. 2): in total, 10,000 events were recorded by flow cytometry (Guava easyCyte5HT, Luminex Corporation) and the resultant data were analyzed by Guava InCyte software (Luminex Corporation). Events were gated according to their FSC/SSC profile (gate R1) to exclude cellular debris. Expression profiles of CD166, CD29, CD105, CD73, CD44 and CD90 were evaluated with respect to isotype control (gate R2).

The following antibodies were used: PE anti-human CD166 antibody (Biolegend, 343,903), PE anti-human CD29 antibody (Biolegend, 303,003), PE anti-human CD105 antibody (Biolegend, 323,205) PE anti-human CD73 (Ecto-5'-nucleotidase) antibody (Biolegend, 344,003), PE anti-human CD44 antibody (Biolegend, 338,807), PE anti-human CD90 (Thy1) antibody (Biolegend, 328,109), PE mouse IgG1, κ isotype control antibody (Biolegend, 400,111), and human BD Fc block, (BD, 564,220).

### Cytokine/chemokine bead assay in vitro

For condition medium (CM) analysis, cells were washed three times with PBS (−), cultured in serum-free α-MEM medium for two days, and used for analysis. Cytokine/chemokine analysis of CM (n = 6) was performed using antibody immobilization on magnetic beads. The CM sample was centrifuged at 13,000*g* and 4 °C for 5 min, and the resulting supernatant was used for measurement. Concentrations of 40 target proteins in CM were measured using a Milliplex® MAP Cytokine/Chemokine Magnetic Bead Panel kit and Luminex® technology (Merck Millipore). Procedures were performed according to the manual (HCYTOMAG-60K.pdf). Pretreated sample was added at 25 μl per well, and measurements were taken three times. Standard solution was prepared as stated in the manual, seven concentrations were prepared in a fivefold serial dilution series, and three measurements (replicates per analysis) were performed. The 41 target proteins were EGF, FGF-2, Eotaxin, TGF-α, G-CSF, Flt-3L, fractalkine, IFNα2, IFNγ, IL-10, GRO,MCP-3, IL-12P40, MDC, IL-12P70, PDGF-AA, IL-13, PDGF-AB / BB, IL-15, sCD40L, IL-17A, IL-1RA, IL-1α, IL-9, IL-1β, IL-2, IL-3, IL-4, IL-5, IL-6, IL-7, IL-8, IP-10, MCP-1, MIP-1α, MIP-1β, RANTES, TNFα, TNFβ, and VEGF (Fig. 3).

### CMAs cell aging study in vitro

Cellular senescence studies on mature adipocytes were conducted to confirm the safety of the CMAs prepared for patients. A characteristic of tumorigenic cells is proliferation without stopping. This test is therefore conducted when the cells are cultured to confirm that cells stop growing. CMAs were cultured until growth arrest was noted for six of the 25 patients. The population doubling level (PDL) was also measured (Fig. 4).

### Clinical application of the CMAs + condition medium and fat graft (CMAM-FGs) in-vivo

A breast-shaped tissue expander was inserted under the pectoralis major muscle and subcutaneously on the affected breast, marked at the location of the healthy breast, and the regular saline solution was injected through the port of the tissue expander, gradually expanding the breast to a size similar to the healthy breast over 6 months. The volume of the healthy breast was defined preoperatively as the breast volume to be achieved during the reconstruction. In the first transplant procedure, half of the saline solution was drawn out from the tissue expander and CMAM-FG was injected around the outside of the capsule of the expander using a cannula with a diameter of 1.2–2.5 mm. In the second procedure, after removal of the expander, the capsule (fibrous tissue) was also removed. The removal of the capsule allows for more blood circulation and improves CMAM-FG engraftment. CMAM-FG was then injected subcutaneously and into the internal cavity created after removal of the expander. The ratio of CMA to autologous fat was determined based on the results of transplantation experiments on nude mice and subsequent partial breast defects and augmentation procedures, which showed ideal engraftment rates at a volume ratio of 1:100. The amount of CMAs for unilateral transplants ranged from 25.74 × 10^7^ to 38.61 × 10^7^ (33.07 × 10^7^ ± 4.29 × 10^7^), and the amount of CMAs for bilateral transplants ranged from 60.06 × 10^7^ to 64.35 × 10^7^ (62.21 × 10^7^ ± 3.03 × 10^7^). Total unilateral injection volumes were 355 to 870 cc (564.52 ± 118.92). Total bilateral injection volumes were 870 to 1336 cc (avg 1103.00 ± 329.51). CM accounted for 20% of the total fat content. Patients with unilateral cases underwent two transplants, and those with bilateral cases underwent three transplants (Table1; Fig. S1). The patient’s BMI was measured pre and post operation and no significant changes were noted. Pre-Op BMI was 22.8 ± 2.5 and Post-Op BMI was 22.4 ± 2.2. No significant difference (P = 0.12) was found. It is also to be noted that the average obtained was similar to the average BMI of Japanese women (40–69 years old) of 22.6 as reported in 2016 the National Health and Nutrition Survey by the Ministry of Health, Labor and Welfare of Japan^48^.

### Immunofluorescence or immunohistochemical analysis of regenerated fat after CMAM-FGs engraftment in-vivo

Biopsies of reconstructed fat six months to one year after transplantation were analyzed histologically. Tissue samples were fixed at 4 °C in 4% PFA, processed, and embedded in paraffin. Transverse sections (4 μm) were placed on MAS-coated slides (Matsunami Glass Ind. Ltd.) for either immunofluorescence or immunohistochemistry.

1. Expression of perilipin as lipid droplet-associated protein.

Anti-perilipin (guinea pig) (Fitzgerald Industries International, 20R-PP004, MA, USA) (4 °C/1 h, 1:200) was used as the perilipin primary antibody, and Alexa Fluor 488 goat anti-guinea pig IgG (InvitrogenA11073, CA, USA) (room temperature/1 h) was used as the secondary antibody. Encapsulation was achieved using Invitrogen™, SlowFade™, Gold Antifade Mountant with DAPI (Sigma-Aldrich) for nuclear staining. Images were examined using a Zeiss LSM510 laser scanning microscope (Fig. 5A).

2. Detection and characterization of macrophages (CD68), angiogenesis (cell surface marker of differentiation: CD31, CD34), and cell proliferation marker (Ki67)^46^.

Primary antibodies were incubated overnight at 4 °C. The primary antibodies used were rabbit monoclonal anti-human CD31 (1:200, Abcam: ab76533), mouse monoclonal CD34 anti-human (1:200, Cell Signal, TEC 3569S), mouse monoclonal CD68 anti-human macrophage (1:500, Abcam, ab955), and rabbit monoclonal anti-human Ki 67(30-9) (Roche, 518102456). After treatment with biotin-labeled secondary antibody (LSAB2, DAKO K0609) at room temperature for 30 min, the sections were stained using DAB (Fig. 5B–E).

### MRI evaluation

MRI images were used to confirm the engraftment of cultured fat. T1 and T2-weighed images were taken in the axial and sagittal sections. Diffusion-weighted images were also taken for the axial section. T2-Fat-Saturation-weighted images were taken in the coronal section.

All patients underwent MRI examinations annually after transplantation, and the images were evaluated by one breast surgeon and two radiologists at other facilities. These evaluations indicated full-layer fat formation for all the patients.

### Statistical analysis

Data are shown as the mean ± SEM from at least six independent experiments. For cytokine/chemokine analysis, the concentration of the target protein (X) was calculated from the calibration curve using Master Plex® QT analytical software (Hitachi Solutions) and the five-parameter logistic function. Functional motion field imaging was used to show median fluorescence intensity.

Statistical analysis using a one-way ANOVA with Dunnett’s test was performed to compare the differences between MCP-1 and other cytokines or chemokines after checking for normality of these data with a Shapiro–Wilk test. Statistical significance was considered as p < 0.01 (two-tailed), and all values are expressed as mean ±SD. Statistical software SPSS Statistics 26 (IBM Corporation, Armonk, NY, USA) was used for these analyses.

### Patient satisfaction scales

The VAS of patient satisfaction is a horizontal line 10 cm long. At the beginning and end are two descriptors representing the minimum and maximum limits of satisfaction (i.e., no satisfaction and total satisfaction)^47^. The patients mark their level of satisfaction on the 10 cm line. For reference there are also five levels of human facial expressions on top of the line.

In the satisfaction survey conducted in September 2023 among patients who had undergone surgery 3–6 years earlier. The scale used was 0–100, 100 expressing the highest level of satisfaction.

### Informed consent

All patients were explained the purpose and scope of this study and they all provided written informed consent to participate in it.

### Supplementary Information

Supplementary Figure 1.

Supplementary Figure 2.

Supplementary Figure 2.

Supplementary Legends.

## Data Availability

All data mentioned in this study is included in this manuscript.
