# Supplementary figures and images for: Successful fat-only whole breast reconstruction using cultured mature adipocytes and conditioned medium containing MCP-1
Source: Sci Rep. 2023 Nov 3;13:18998. doi: 10.1038/s41598-023-45169-1 (PMC10624668; doi:10.1038/s41598-023-45169-1)

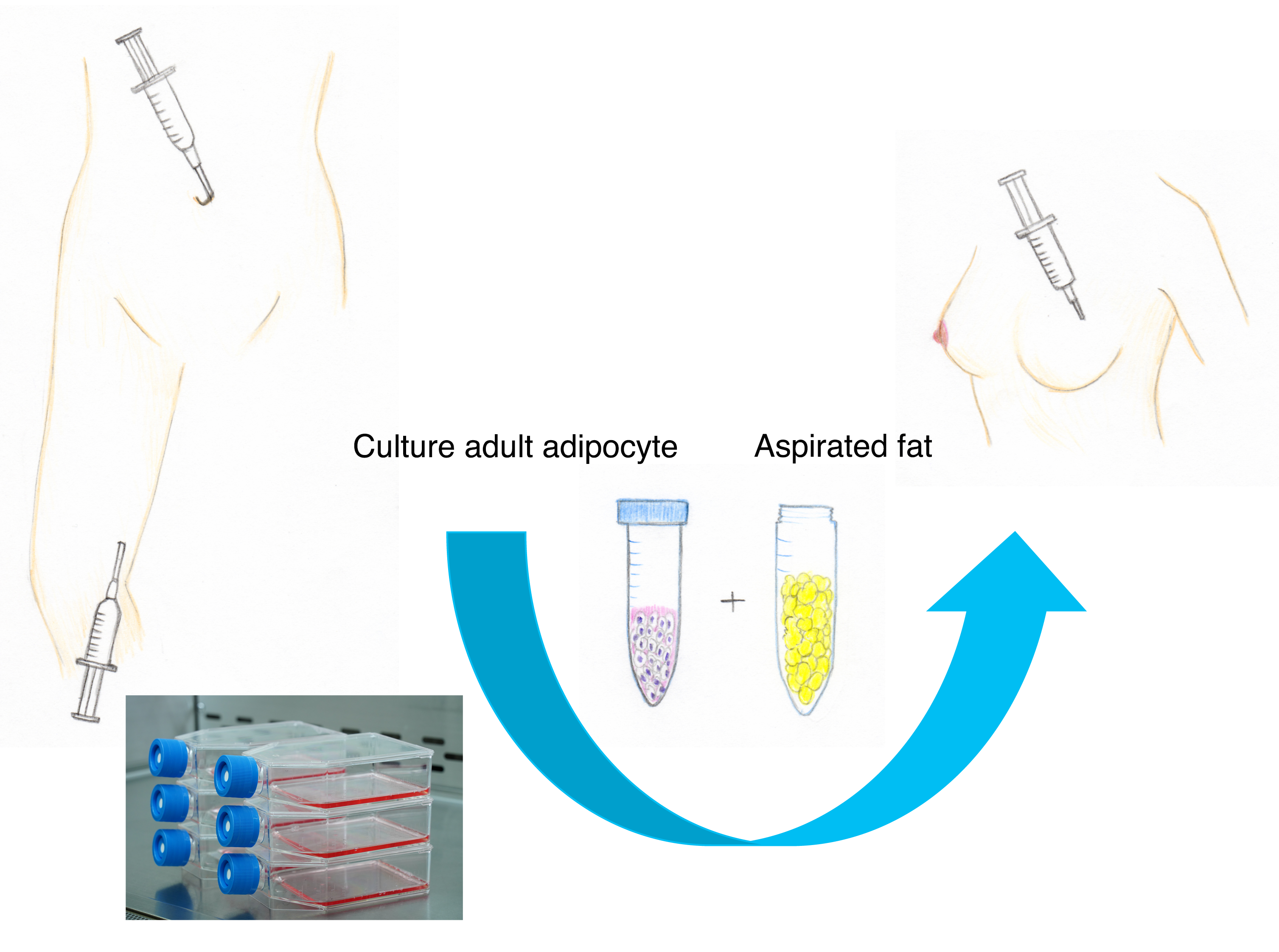

Supplement: Supplementary file 1 — Supplementary Figure 1. [file 41598_2023_45169_MOESM1_ESM.tif]

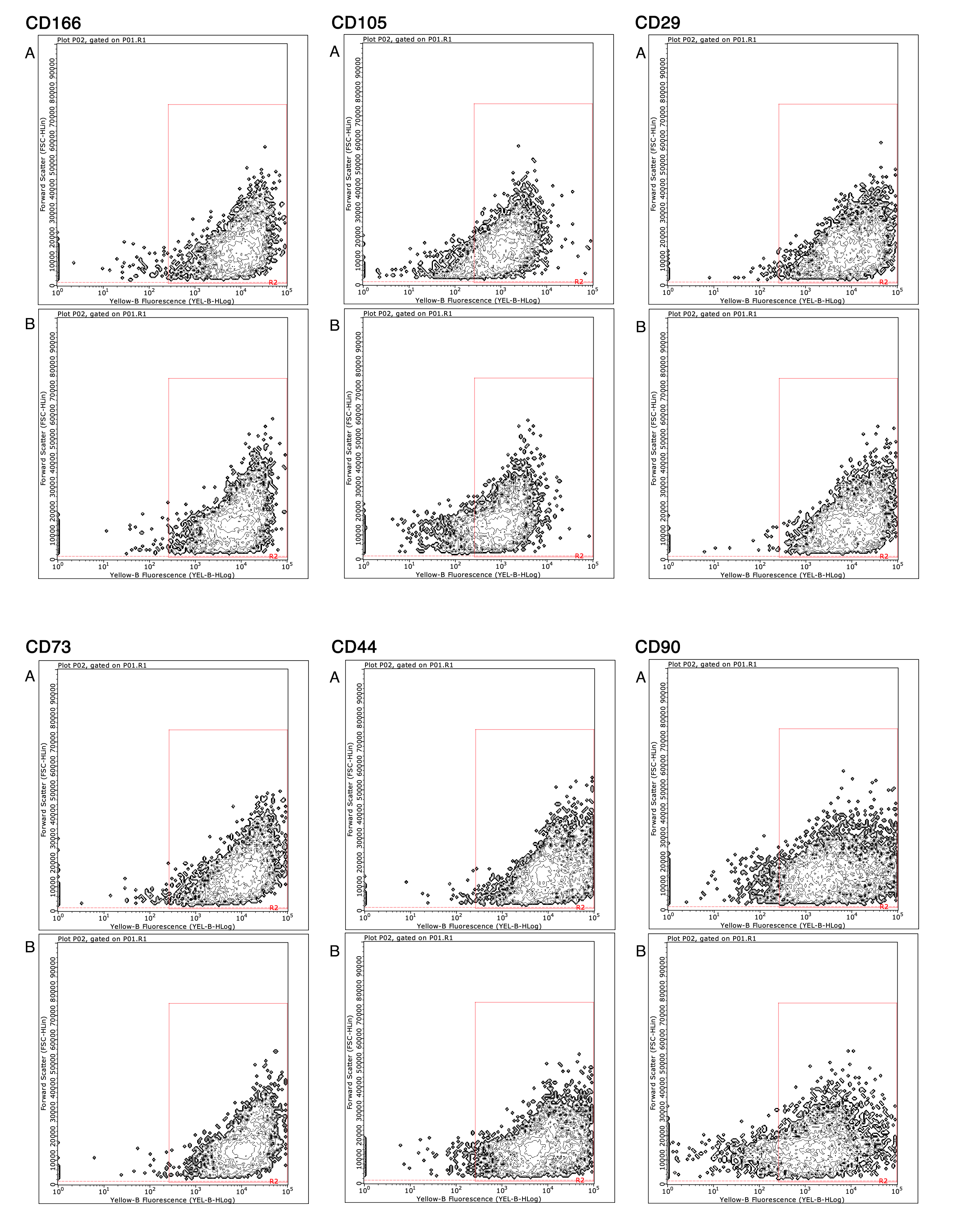

Supplement: Supplementary file 2 — Supplementary Figure 2. [file 41598_2023_45169_MOESM2_ESM.jpg]

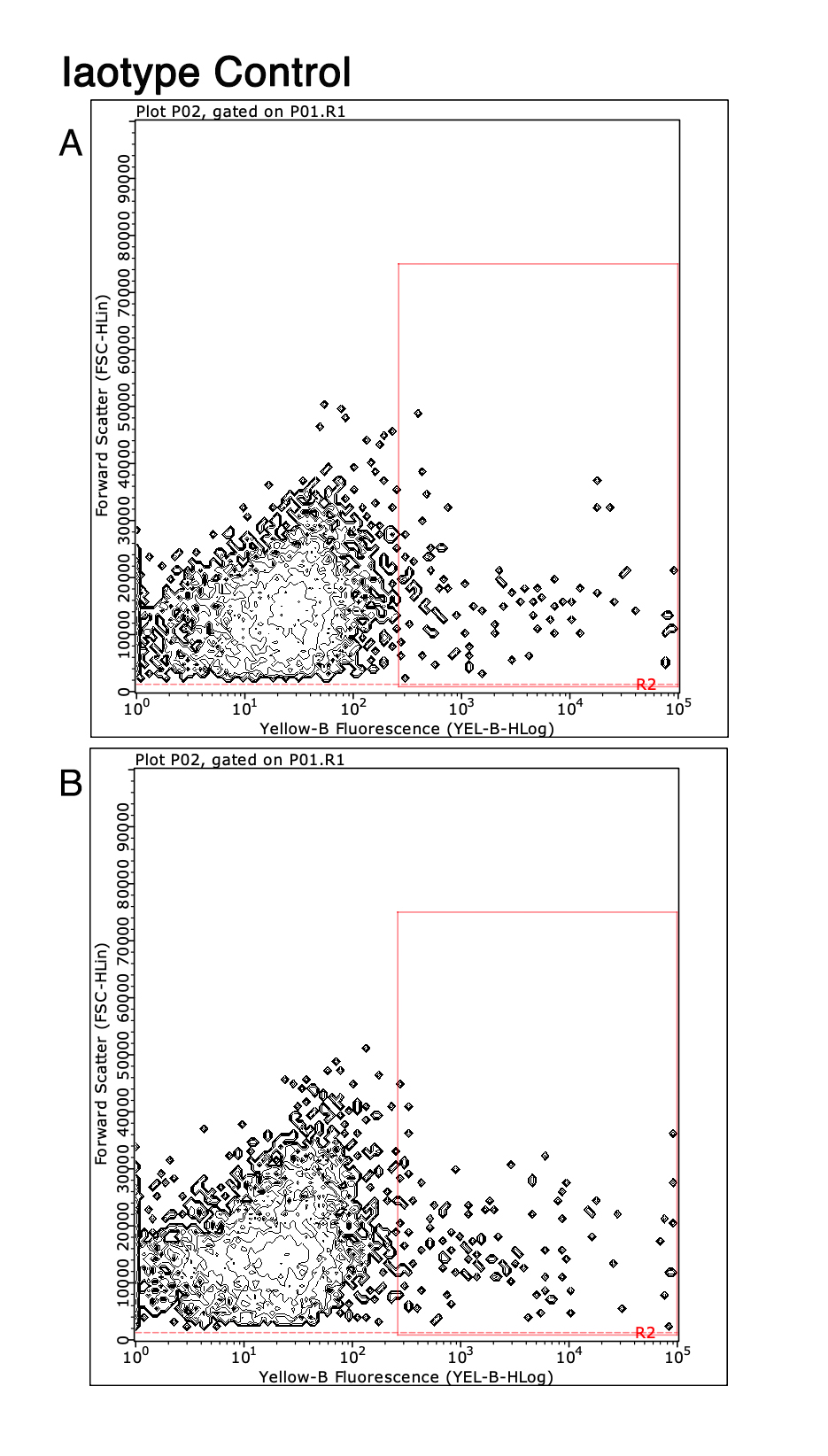

Supplement: Supplementary file 3 — Supplementary Figure 2. [file 41598_2023_45169_MOESM3_ESM.jpg]
