# Supplementary material for: Successful fat-only whole breast reconstruction using cultured mature adipocytes and conditioned medium containing MCP-1
Source: Sci Rep. 2023 Nov 3;13:18998. doi: 10.1038/s41598-023-45169-1 (PMC10624668; doi:10.1038/s41598-023-45169-1)
Supplement: Supplementary file 4 — Supplementary Legends. [file 41598_2023_45169_MOESM4_ESM.docx]

**Supplementary figure legends**

**Figure S1. Schema of transplantation of human mature adipocyte culture + condition medium and autologous aspirated fats**

About 2 cc of subcutaneous fat from the lower abdomen or thighs was aspirated, expanded by increasing mature adipocytes, mixed with autologous aspirated fat + conditioned medium, and transplanted by injection into the chest area.

**Figure S2.** Contour plots with outliers of flow cytometry analysis of the expression of cell surface markers related to various stem cells on SVFs (left) and CMAs (right). IC: Isotype control antibodies were used for control samples. CMAs showed higher expression levels of CD44 and CD90 than observed in SVFs.
